# Inflammatory, Hematological, and Biochemical Biomarkers in COVID‐19 Patients

**DOI:** 10.1002/iid3.70078

**Published:** 2024-12-06

**Authors:** Rebeccah M. Ayako, Kirtika Patel, Isaac Ndede, Johan Nordgren, Marie Larrson, Simeon K. Mining

**Affiliations:** ^1^ Department of Pathology Moi University Eldoret Kenya; ^2^ Department of Biomedical and Clinical Sciences, Division of Molecular Medicine and Virology Linköping University Linköping Sweden

**Keywords:** biochemical, COVID‐19 disease severity, hematological, inflammatory biomarkers

## Abstract

**Introduction:**

There are few accurate prognostic indications of the illness's development and severity for COVID‐19, despite certain biomarkers having been investigated. The unexpected nature of COVID‐19's course, which can quickly progress from asymptomatic to life‐threatening symptoms, lies at the heart of the disease's intricacy. Predicting SARS‐CoV‐2 pathogenicity through laboratory biomarkers and as such, identifying the patients’ illness severity at the time of their initial admission would be crucial in improving patient care. In this study, we sought to evaluate the potential of hematological, biochemical, and inflammatory biomarkers in predicting the course of COVID‐19 at a tertiary hospital in western Kenya.

**Methods:**

This cross‐sectional study involved 48 COVID‐19 patients (16 asymptomatic; 16 moderate symptomatic; and 16 severe symptomatic) and 48 age‐sex‐matched COVID‐19‐negative clients attending the Moi Teaching and Referral Hospital, Kenya. Demographic information, self‐reported chronic illnesses, symptoms, and laboratory results were collected at recruitment.

**Results:**

Significantly, the severity of COVID‐19 was associated with; hemoglobin (*p* < 0.0001), white blood cells (*p* = 0.0022), hematocrit (*p* < 0.0001), blood urea nitrogen (*p* = 0.01), blood sodium (*p* = 0.0002), potassium (*p* = 0.0483), C‐reactive protein (*p* = 0.0002), and Lactate Dehydrogenase (*p* < 0.0001). Regression analysis of CRP revealed a strong positive correlation (*p* = 0.0006) whereas LDH revealed a weak positive correlation (*p* < 0.0001) with COVID‐19 disease severity. Discriminative accuracy was highest when asymptomatic was compared to severe COVID‐19 for CRP and LDH (AUC: 0.8867, 95% CI: 0.7532–1.000) and (AUC: 1.000, 95% CI: 1.000–1.000) respectively.

**Conclusion:**

The hematological indices, inflammatory and biochemical biomarkers studied have the potential to predict the course of COVID‐19. These parameters may be useful in helping design appropriate care for COVID‐19 patients.

## Graphical abstract

Inflammatory biomarkers are significantly elevated in severe COVID‐19 patients. These biomarkers may be used to predict COVID‐19 severity upon admission.

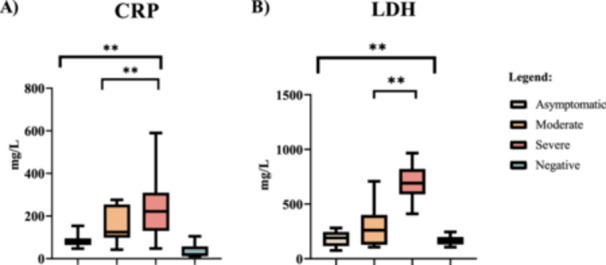

## 1 Introduction

The first case of severe acute respiratory syndrome coronavirus 2 (SARS‐CoV‐2), was discovered in Wuhan, China, in December 2019. The World Health Organization designated COVID‐19 as a Public Health Emergency of International Concern in March 2020. As of April 13, 2024, COVID‐19 had spread to every country in the world, resulting in more than 704,753,890 COVID‐19 cases and 7,010,681 fatalities. Most counties reported COVID‐19 incidences within 40 days of the WHO declaration of the COVID‐19 pandemic. In Africa, about 5,382,790 COVID‐19 cases and 140,682 deaths are on record [1]. On March 13, 2020, Kenya declared the first COVID‐19 case. Since then, Kenya recorded 344,130 COVID‐19 cases as of September 27, 2023, with 5689 deaths. During the study period, the Kenya COVID‐19 vaccination status rose to about 30.7% of eligible people aged ≥ 18 years as was then recommended by WHO and the Ministry of Health Kenya [2].

At the time, in COVID‐19 patients with pneumonia, 20%–30% progressed to respiratory failure necessitating intubation and ventilator assistance [3]. Due to the COVID‐19 pandemic's fast spread, the afflicted nations adopted diverse and dynamic methods to identify those in need of urgent care [4]. SARS‐CoV‐2 nucleic acid detection by real‐time reverse transcription polymerase chain reaction (RT‐qPCR) in respiratory specimens, such as sputa, oro‐nasopharyngeal swabs, and nasal aspirates, is currently the gold standard for the diagnosis of COVID‐19 patients [5]. But its availability, cost and longer turnaround time are major drawbacks in resource‐limited countries.

The clinical symptoms of COVID‐19 range from asymptomatic to severe respiratory distress [6]. The first studies treated COVID‐19 as if it were merely pneumonia but correlated data have shown that intravascular coagulation and coagulopathy are frequent in individuals with high SARS CoV‐2 viral load, leading to an increase in fatality rate [7, 8] observed that clinical hematology, inflammatory, and biochemical laboratory findings are important in the triage and may offer helpful prognoses for COVID‐19 patients. He showed that the majority of COVID‐19 patients have abnormal pro‐calcitonin (PCT), reduced white blood cell and lymphocyte count, neutrophilia, thrombocytopenia, elevated C‐reactive protein (CRP), and erythrocyte sedimentation rate (ESR). These changes could help predict the severity and stage of the illness [9].

This study aimed to investigate hematological, inflammatory, and biochemical biomarkers in COVID‐19‐positive individuals and their relationship with disease severity among COVID‐19 patients at Moi Teaching and Referral Hospital (MTRH) in western Kenya. Our study results also help point out factors associated with COVID‐19 progression into severe disease. This knowledge is expected to inform preventive and curative interventions.

## 2 Materials and Methods

### 2.1 Study Design

This was a cross‐sectional hospital‐based study of 48 COVID‐19‐positive patients visiting the MTRH based in Eldoret, Kenya and 48 COVID‐19‐negative study volunteers recruited as controls between May 2022 to February 2023. The Omicron (BA.5‐XBB.1.5) SARS‐CoV‐2 strain was the circulating virus strain in Kenya at the time. The studied COVID‐19 patients were stratified into three categories by CDC criteria: (1) asymptomatic disease (needing no medical attention, *n* = 16); (2) moderate disease (needing hospitalization but not intensive care, *n* = 16); and (3) severe disease (requiring intensive care, *n* = 16) [10]. SARS‐CoV‐2 negative individuals were healthy were age and sex matched the positive participants.

### 2.2 Ethical Approval

This study was approved by the Institutional Research and Ethics Committee of the Moi University/MTRH Eldoret, Kenya (FAN 0004608). After a clear explanation of the study's objectives, prospective participants were asked to provide written informed consent before being recruited into the study.

### 2.3 Study Site and Population

This study was conducted at MTRH, Kenya among COVID‐19‐positive and COVID‐19‐negative volunteers/travellers who served as healthy controls at MTRH.

#### 2.3.1 Sample Size and Sampling

The study sample size was calculated using G*Power Sample Size software version 3.1.9.7 applying variables from a similar study by Wang [11]. From this study and using a mean CRP level in the mild COVID‐19 group of 1.52 ± 1.56 relative to the moderate 16.76 ± 18.38, severe 54.15 ± 1.06 and critical 105.00 ± 12.73 COVID‐19 groups, a total sample size of 48 (16 per group) were required to detect a meaningful effect size of 0.2766 with a 99% power leaving a 1% risk of a Type II error, which is the failure to identify that valid effect at 0.05 significance level using a one‐way analysis of variance (ANOVA) for asymptomatic, moderate and severe patients.

The study employed a purposive sampling technique. Symptomatic patients were recruited before admission to the wards and the COVID‐19 Isolation Center in the Hospital. Asymptomatic participants were recruited from travellers who tested positive for SARS‐CoV‐2 at the MTRH COVID‐19 testing facility. The first participant who met the inclusion criteria was recruited and thereafter every third COVID‐19‐positive patient and COVID‐19‐negative fifth traveler was recruited in the study until 48 participants were recruited.

#### 2.3.2 Inclusion‐Exclusion Criteria

COVID‐19 cases were included if they fulfilled the following three (3) criteria: Clinical symptoms as verified by a physician, positive RT‐qPCR SARS‐CoV‐2 test, and capacity to participate as determined by the ability to consent. The COVID‐19 negative group was negative for the RT‐qPCR SARS‐CoV‐2 test with a history of underlying conditions similar to the positive participants.

For both the case and control groups, COVID‐19‐vaccinated individuals, expectant women, and individuals with certain chronic illnesses such as HIV and TB were excluded from the study since these infections would affect their hematological and inflammatory parameters. Noncommunicable chronic illnesses such as hypertension, diabetes, asthma, cardiac, kidney, and liver diseases were noted.

### 2.4 Laboratory Tests

A hematology analyzer (MINDRAY BC 6800 Beijing, China), was used to determine hematological parameters including complete blood count. To determine LDH and CRP inflammatory biomarkers, the VITROS 5600/XT 7600 (Integrated Systems USA) was used per the manufacturer's recommendations.

### 2.5 Statistical Analysis

The results from the laboratory were entered into the GraphPad Prism version 9.0 (GraphPad Software, La Jolla, CA, USA). Categorical variables have been presented as either mean with standard deviation (SD) for parametric and median with IQR for nonparametric. Test for normality was done using Kolmogorov–Smirnov with Dallal–Wilkinson–Lillie. ANOVA test with a Tukey post‐hoc test was used for normally distributed data while the Kruskal–Wallis followed by post‐hoc Dunn's test was employed in nonnormally distributed data. Mann Whitney *U*‐test was used to analyze nonnormally distributed data while a paired *t*‐test was used to compare continuous variables for normally distributed data. Regression analysis results are presented as adjusted odds ratios (aOR) and 95% confidence intervals (CI). The optimal cut‐off point of the analyzed markers in detection was determined by evaluating prediction accuracy using the area under the curve, the receiver operating characteristic (ROC) curve. Using a sensitivity/specificity versus criteria value plot, the cut‐off values with the highest accuracy were identified. The cut‐off for significance was set at *p* < 0.05.

## 3 Results

Of the 16 asymptomatic individuals recruited into the study, nine were male and seven were female. The moderate individuals had eight males and eight females. Among the severe COVID‐19 cases were 11 males and five females. All the recruited individuals were between 20 and 72 years of age, the median age was 35.5 years IQR 29–50 years. The COVID‐19 negative control groups were age and sex‐matched with the COVID‐19 positive cases (Table 1).

**Table 1:** Characteristics of COVID‐19 patients.

Measures	Asymptomatic disease *n* = 32		Moderate disease *n* = 32		Severe disease *n* = 32		Statistic	*p*‐value
	Case	Control	Case	Control	Case	Control		
	*n* = *16*	*n* = 16	*n* = *16*	*n* = *16*	*n* = *16*	*n* = *16*		
Demographics								
Age—years; median (IQR)	46 (29–56.5)	45 (29.5–55)	39 (32.5–46)	37.5 (32–45.5)	32 (27–45.5)	42 (26.5–58)	0.4202**	0.1722
Female; *n* (%)	7 (44)	7 (44)	8 (50)	8 (50)	5 (31)	5 (31)	1.2*	0.5488
Vital signs								
Body temperature (°C); median (IQR)	36.4 (36.3–36.8)	36.35 (36.2–36.4)	36.5 (35.35–36.8)	36.5 (36.4–36.35)	36.65 (36.4–36.8)	36.8 (36.4–36.8)	0.0001**	0.7136
Systolic (mm/Hg); mean ± SD	129.8 ± 19.97	130.8 ± 17.79	114.7 ± 11.55	127.4 ± 16.47	139.8 ± 29.12	128.4 ± 14.86	5.675***	0.0061
Diastolic (mm/Hg); mean ± SD	81.81 ± 11.46	82.75 ± 9.04	71.73 ± 14.46	78.56 ± 14.63	78.79 ± 18.00	74.37 ± 13.28	2.145***	0.1281
Oxygen saturation (%); mean ± SD	94.31 ± 2.86	94.56 ± 2.52	92.51 ± 4.37	93.34 ± 4.37	79.11 ± 11.59	94.58 ± 2.26	22.91***	< 0.0001

* Chi‐square,

** *H*‐statistic,

*** *F*‐statistic.

Patients with severe COVID‐19 had a higher frequency of comorbid illnesses than moderate symptomatic and asymptomatic; hypertension (25% vs. 12.5% vs. 12.5%), pulmonary disease (25% vs. 25% vs. 12.5%), chronic kidney disease (31.25% vs. 12.5% vs. 6.25%), cardiac disease (25% vs. 18.75% vs. 18.75%), asthma (43.75% vs. 25% vs. 6.25%), diabetes (50% 37.5% vs. 18.75%) and liver diseases (50% vs. 37.5% vs. 50%) (Table 2)**.**

**Table 2:** Comorbidities of SARS‐CoV‐2 infected patients.

Measures	Asymptomatic disease *n* = 16		Moderate disease *n* = 16		Severe disease *n* = 16		*p*‐value
	Frequency		Frequency		Frequency		
	(*n*)	(%)	(*n*)	(%)	(*n*)	(%)	
Cardiac disease (%)	3	18.75	3	18.75	4	25	0.0099*
Hypertension (%)	2	12.5	2	12.5	4	25	0.0572
Pulmonary disease (%)	2	12.5	4	25	4	25	0.0377*
Asthma (%)	1	6.25	4	25	7	43.75	0.2143
Kidney disease (%)	1	6.25	2	12.5	5	31.25	0.1567
Liver disease (%)	8	50	6	37.5	8	50	0.0059*
Diabetes (%)	3	18.75	6	37.5	8	50	0.0059*

*p < 0.05.

Our results show a significant association between hematological parameters and severe outcomes. In this study, asymptomatic COVID‐19 patients had a higher mean WBC count (5.697 ± 1.617 × 10^9^/L) than the moderate (5.525 ± 1.652 × 10^9^/L), and severe (4.736 ± 1.253 × 10^9^/L) groups. Severe symptomatic individuals had significantly lower WBC as compared to the asymptomatic individuals (*p* = 0.0224). Mean hemoglobin levels in severe symptomatic individuals were significantly lower (7.27 ± 1.354 × 10^9^/L) than in asymptomatic (11.38 ± 3.397 × 10^9^/L) and moderate symptomatic patients (11.54 ± 2 × 10^9^/L) (*p* < 0.0001).

**Figure 1:**
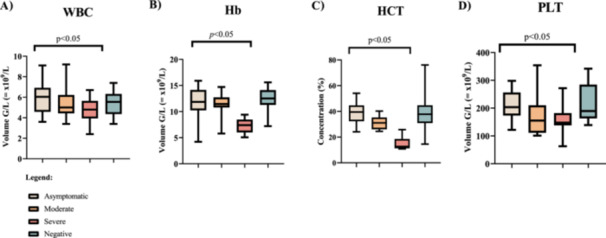
Hematological indices in COVID‐19 positive and COVID‐19 negative individuals. White blood cells (A: WBC), hemoglobin (B: Hb), hematocrit (C: HCT), and platelets (D: PLT).

The mean HCT levels were significantly lower, in the severe COVID‐19 patients (14.67 ± 4.512%) in contrast to asymptomatic (38.84 ± 8.227%) and moderate symptomatic individuals (30.88 ± 4.803%) (*p* < 0.0001). Pairwise comparison analysis using the Mann–Whitney *U*‐test also revealed significant HCT differences between asymptomatic disease and moderate disease (*p* = 0.0022), asymptomatic disease and severe disease (*p* < 0.0001), and moderate versus severe disease (*p* < 0.0001). Our data show thrombocytopenia in severe COVID‐19 patients median 95% CI (IQR) 147.5 (131–189 × 10^9^/L) when compared to patients with moderate disease median 95% CI (IQR) 154.85 (113.5–206.5 × 10^9^/L) and asymptomatic disease median 95% CI (IQR) 203.5 (176–252 × 10^9^/L). Mann–Whitney *U*‐test revealed significant differences between asymptomatic disease with severe disease (*p* = 0.0249). Analysis of PLT data using Kruskal–Wallis revealed significant differences in the PLT levels among the asymptomatic, moderate symptomatic, and severe symptomatic cases (*p* = 0.0418) (Figure 1).

**Figure 2:**
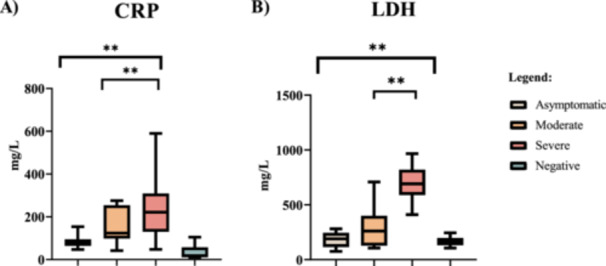
Inflammatory biomarker in COVID‐19‐positive and COVID‐19‐negative individuals. C‐reactive protein (H: CRP) and lactate dehydrogenase (I: LDH) are inflammatory biomarkers. **p < 0.05.

Analysis using Mann–Whitney *U*‐test analysis of inflammatory indicator's data shows significant CRP differences between asymptomatic illness and moderate disease (*p* = 0.0037), asymptomatic disease and severe disease (*p* = 0.0001), and moderate and severe disease (*p* = 0.0041). When compared to the asymptomatic (86.31 ± 28.21 mg/L) and moderate symptomatic group median 95% CI (IQR) 124.5 (99.5–249.5 mg/L), severe symptomatic (241.5 ± 138.8 mg/L) had significantly higher CRP levels (*p* < 0.0001). Similarly, mean LDH levels in severe symptomatic patients were significantly higher 694.3 ± 159.1 mg/L while moderate symptomatic had 297.9 ± 178.8 mg/L, and asymptomatic had 184.6 ± 62.83 mg/L (*p* < 0.0001) (Figure 2). There were significant differences in LDH between asymptomatic disease and moderate disease (*p* = 0.0232), asymptomatic disease and severe illness (*p* < 0.0001), and moderate versus severe disease (*p* < 0.0001) (Figure 2).

Further, high blood urea nitrogen (BUN) was recorded in severe patients median 95% CI (IQR) 49.5 (41.75–69.75 mmol/L) while individuals with moderate disease had (48.01 ± 12.88 mmol/L) and asymptomatic disease (39.36 ± 9.103 mmol/L). Significant differences were observed between severe with asymptomatic disease (*p* = 0.0037) and asymptomatic disease with moderate disease (*p* = 0.0361). In comparison, Kruskal–Wallis and Dunn's post‐hoc test showed significant BUN levels between the groups (*p* = 0.01).

Significant mean blood sodium differences between moderate with severe disease and severe with asymptomatic disease were observed (*p* = 0.0002 and *p* = 0.0052, respectively). Significant hyponatremia (*p* = 0.0003), analyzed using Kruskal–Wallis, was observed in severe patients where they had a mean of 116.4 ± 14.24 mmol/L of sodium, moderate symptomatic patients had 134.4 ± 19.13 mmol/L while the asymptomatic patients had a median of 136.5 and IQR values of 131–151.85 mmol/L respectively.

**Figure 3:**
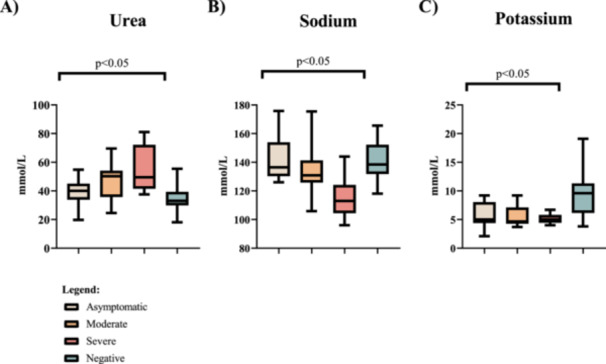
Biochemical markers in COVID‐19 positive and COVID‐19 negative. Urea: (A) is a marker of the kidney function test. Sodium (B) and potassium (C) are markers of serum electrolytes.

Severe patients recorded significant hypokalemia (5.138 ± 0.7658 mmol/L) while asymptomatic median 95% CI (IQR) five (4.5–7.97 mmol/L) and moderate patients median 95% CI (IQR) 4.65 (4.35–7 mmol/L) (*p* = 0.028). Asymptomatic sickness and moderate disease (*p* = 0.0396), asymptomatic disease and severe disease (*p* = 0.0294), and moderate versus severe disease (*p* = 0.0185) were demonstrated to have significant discrepancies in blood potassium levels using Mann–Whitney *U*‐test analysis (Figure 3).

Our results show a significant association between hematological, inflammatory, and biochemical biomarkers with COVID‐19 severity. On regression, our analysis revealed an association between WBC (OR: 6.144 [−0.9537, 0.1367], *p* = 0.0032), Hb (OR: 0.5964 [−0.5370, 0.4521], *p* = 0.0017), HCT (OR: 0.979 [−2.082, 1.947], *p* = 0.0008) and PLT (OR: 5.0263 [0.5013−1.2688] *p* < 0.0001). Regarding inflammatory biomarkers, there was also an association between CRP (OR: 2.581 [9.361−110.9], *p* = 0.0006) and LDH (OR: 0.2367 [−0.6948, 1.252], *p* < 0.0001) with disease severity. Regression analysis showed an association between BUN (OR: 1.923 [−0.032, 0.5431], *p* = 0.0191), blood sodium (OR: 4.4133 [−0.3521, 0.6588], *p* = 0.0005) and blood potassium (OR: 2.3952 [−1.6570, 2.3177], *p* < 0.0001) (Table 3).

**Table 3:** Logistic regression analysis of inflammatory biomarkers and disease severity.

Inflammatory biomarkers	Standard error	OR (95% CI)	*p*‐value
Hematological indices			
WBC	0.3138	6.144 (–0.9537, 0.1367)	0.0032
Hb	0.1535	0.5964 (−0.5370, 0.4521)	0.0017
HCT	0.4811	0.979 (−2.082, 1.947)	0.0008
PLT	0.1761	5.0263 (0.5013, 1.2688)	< 0.0001
Inflammatory biomarkers			
CRP	0.0395757	2.581 (9.361, 110.9)	0.0006
LDH	0.176826	0.2367 (−0.6948, 1.252)	< 0.0001
Biochemical markers			
BUN	0.1342	1.923 (−0.032, 0.5431)	0.0191
Sodium	0.2356	4.4133 (−0.3521, 0.6588)	0.0005
Potassium	0.6096	2.3952 (−1.6570, 2.3177)	< 0.0001

**Figure 4:**
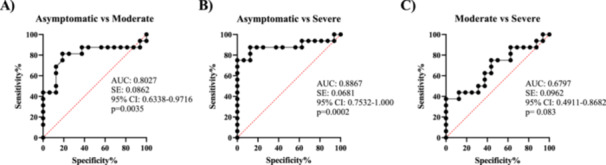
Receiver operating characteristic (ROC) curves of CRP for predicting COVID‐19 severity. (A: asymptomatic vs. moderate disease), (B: asymptomatic vs. severe disease), and (C: moderate vs. severe disease).

The CRP and LDH inflammatory biomarkers were further assessed for their ability to predict COVID‐19 severity. The ROC of CRP in asymptomatic versus severe disease revealed the AUC was 0.8867, 95% CI between 0.7532 and 1.000, *p* = 0.0002. In the case of asymptomatic versus moderate disease, the AUC was 0.8027, *p* = 0.0035% and 95% CI between 0.6338 and 0.9716 with a sensitivity of 87% and specificity of 94%. In moderate versus severe disease, the ROC‐AUC was 0.6797, *p* = 0.083 with a 95% CI between 0.4911 and 0.8682 (Figure 4).

**Figure 5:**
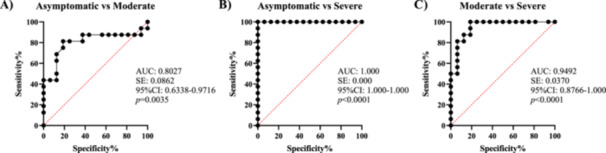
Receiver operating characteristic (ROC) curves of LDH for predicting COVID‐19 severity. (A: asymptomatic vs. moderate disease), (B: asymptomatic vs. severe disease) and (C: moderate vs. severe disease).

Regarding LDH, ROC‐AUC was 1.0 with a 95% CI between 1.0 and 1.0, *p*‐value < 0.0001. With a sensitivity of 100% and a specificity of 100% between asymptomatic and severe disease, 142.75 mg/L was shown to be the best cut‐off value as a predictor of severe illness. The ROC‐AUC values between asymptomatic versus moderate were 0.9492 and a 95% CI between 0.8766 and 1.0, *p* < 0.0001. In asymptomatic versus severe disease, the AUC was 0.9492 with a 95% CI between 0.8766 and 1.0, *p* < 0.0001 (Figure 5).

## 4 Discussion

This study evaluated relationships between inflammatory, hematological, and biochemical biomarkers, with clinical characteristics in asymptomatic, moderate, and severe COVID‐19 outcomes. Based on the present study, relatively younger individuals with median (IQR) 32 (27–45.5) had severe disease as compared to those with median (IRQ) 39 (32.5–46) with moderate and 46 (29–56.5) who were asymptomatic COVID‐19 participants. This may be attributed to selection bias in patient recruitment in that individuals older than 65 years were not recruited in the study as they had been vaccinated against COVID‐19. We report incidences of comorbidities in this age range. This is supported by recent studies in the study region reporting changing lifestyles among younger individuals which may influence the incidence of comorbidities in this segment of the population [12, 13]. On the contrary, patients with COVID‐19 who are older have a higher chance of developing serious illness due to age‐related comorbidities. The physiological aging process and, more specifically, the higher incidence of frailty and comorbidities in older adult patients, which lead to a decline in functional reserve that lowers intrinsic capacity and resilience and makes it more difficult to fight off infections, may have an impact on this fact. According to this theory, comorbid conditions including diabetes, hypertension, and cardiovascular disease are quite common in older persons and have been linked to worse COVID‐19 results.

We found that COVID‐19 individuals' Hb and HCT levels were considerably lower than the asymptomatic, moderate, and COVID‐19 negative groups. This result is consistent with Peng et al. [14] and Tiwari et al. [15] who found dramatically reduced Hb/HCT in severe and critically ill COVID‐19 patients. Low hemoglobin and hematocrit levels increase the risk of acute respiratory distress syndrome (ARDS), heart anomalies, and kidney damage by impairing the body's capacity to satisfy oxygen needs [16]. Individuals with COVID‐19 who have lower hematocrit levels are more likely to experience multiorgan dysfunction, require longer ICU stays, and die [17]. These parameters are more common in anemic individuals, implying that inflammation is a likely pathophysiological process causing the anemia in that thrombotic microangiopathy has also been implicated as the underlying cause of anemia in COVID‐19 patients with a decreased platelet count [18]. Although the precise mechanism by which COVID‐19 induces anemia is not entirely known, it has been proposed that it could do so by preventing erythropoiesis in the bone marrow [19, 20]. Regular monitoring of hematological parameters including hemoglobin and hematocrit levels in COVID‐19 patients. A drop in hemoglobin or hematocrit levels may indicate the need for erythropoietin treatment, oxygen supplementation, and blood transfusions and should warn physicians of the potential for severe hypoxia, systemic inflammation, or organ malfunction common in severe COVID‐19 patients [21]. These parameters may have prognostic value especially those with signs of severe illness.

In our study, WBC increased from negative, asymptomatic to moderate and to severe group. Previous research has indicated that mild to moderate COVID‐19 patients frequently have normal or slightly decreased white blood cell counts. Leukopenia, on the other hand, is more common in individuals who are critically unwell [22]. Additionally, Qin et al. [23] investigated signs of immune response dysregulation in 452 individuals with confirmed COVID‐19 and reported that severe cases had greater leukocyte counts than the mild group. According to a retrospective analysis of COVID‐19 patients, low WBC counts were found in about 25% of them, and they were linked to a more aggressive course of the illness [24]. Henry et al. [25] also found that the WBC was significantly greater in patients with severe and fatal illnesses compared to nonsevere patients in a meta‐analysis of 21 studies of 3377 COVID‐19‐positive individuals. This observation may be related to cytokine storm and strong inflammatory reactions in COVID‐19 leading to WBC hyperplasia and/or hyperactivation, which can lead to consequences such as ARDS [26]. Leukocytosis suggests an overactive immunological response that can cause tissue damage and conditions including ARDS. Periodic WBC count monitoring is crucial from a clinical standpoint for spotting severe disease development early [27]. Clinicians should be made aware of the potential for cytokine storm and the necessity of early anti‐inflammatory therapies such as corticosteroids or cytokine inhibitors such as anti‐IL‐6 in patients with elevated WBC counts.

We also observed a decrease in platelet counts in severe COVID‐19 patients. About 5%–10% of COVID‐19 patients had decreased platelet counts compared to negative individuals [28]. Lower counts are frequently associated with worse clinical outcomes [29]. According to studies, thrombocytopenia is very prevalent in COVID‐19 patients, particularly in more severe instances [30, 31]. In a meta‐analysis, COVID‐19 non‐survivors often had far lower platelet counts than survivors [28]. Thus, the severity of thrombocytopenia can also be utilized as a predictor of outcome. According to one study, the incidence of severe COVID‐19 increased dramatically when the platelet count dropped by 50 × 10^9^/L [32]. These results align with Lippi, Plebani, and Henry [32] who found that a decline in PLT count (23 × 10^9^/L to 31 × 10^9^/L) was associated with an increased risk of mortality in hospitalized individuals. Platelet count correlation with illness severity and mortality risk in the intensive care unit (ICU) makes it a possible biomarker to predict COVID‐19 disease severity.

In this study, severe patients had higher CRP levels (241.5 ± 138.8 mg/L) in comparison to the asymptomatic (86.31 ± 28.21 mg/L) and moderate symptomatic group median 95% CI (IQR) 124.5 (99.5–249.5 mg/L). Critically sick individuals frequently have readings above 150 mg/L [29]. Furthermore, consistently elevated CRP levels are linked to poorer outcomes and longer hospital admissions [34]. Similarly, patients with mild or moderate illness, and individuals with severe pneumonia, ARDS, or multiorgan failure typically have significantly higher CRP levels [35]. CRP levels in patients with severe COVID‐19 were more than three times greater than those in moderate instances, according to a meta‐analysis [36]. Studies have consistently shown that higher CRP levels are associated with worse clinical outcomes of many diseases often requiring the need for intensive care, and mechanical ventilation, and with increased chances of mortality [37, 38]. The liver produces considerable amounts of acute‐phase proteins (APPs), including CRP, in response to infections including SARS‐CoV‐2 [39]. Thus, acute inflammatory proteins like SARS‐CoV‐2 correlate well with infection, inflammation, and tissue damage occasioned by the virus [40]. Thus, CRP levels may therefore be a biomarker of COVID‐19 disease severity and predictor for the progression of COVID‐19 patients to severe disease in our study setting. The ability of CRP to differentiate between mild and severe illness courses was further supported by the much lower CRP levels seen in asymptomatic and moderately afflicted individuals. This differential is therapeutically significant because more active monitoring and action may be necessary to improve patient outcomes if increased CRP levels are detected early.

Our study shows that the levels of LDH are substantially higher in severe individuals (694.3 ± 159.1 mg/L) in contrast to the moderate (297.9 ± 178.8) and asymptomatic patients (184.6 ± 62.83). Several studies have linked increases in LDH levels above specific thresholds (e.g., > 250 U/L) to an increased risk of unfavorable outcomes, including mortality [41, 42]. Tissue damage brought on by viral replication alongside immune‐mediated injury, as well as hypoxic circumstances typical of severe COVID‐19, especially in patients with pneumonia and ARDS, are likely the causes of the high rise of LDH in severe COVID‐19 cases [43]. In extreme situations, an elevated level of LDH may indicate multiorgan involvement and widespread systemic inflammation. Interstitial lung infections and acute and severe lung damage have both been linked to an increase in LDH levels [44]. When a chest CT scan was used to validate the prediction, an increase in LDH of 62.5 U/L had a good sensitivity and a high specificity indicating a considerably increased likelihood of disease progression [45]. In another study, CRP and LDH were shown to be quite strong independent predictors: COVID‐19‐related mortality, the WHO clinical progression scale, and the degree of illness [46]. In the therapeutic care of COVID‐19 patients, this lends credence to the idea that LDH might be a helpful predictive tool by acting as a proxy marker for the degree of tissue damage.

In the current study, severe COVID‐19 patients had considerably higher (49.5 (41.75–69.75 mmol/L) BUN, renal indicator, at admission. Our results suggest a role of higher BUN, at admission as a predictor of unfavorable outcomes in COVID‐19 patients. Increased urea levels have also been reported in many studies of patients with severe COVID‐19. Zhang and Ma [47] found that non‐survivors of COVID‐19 had considerably greater urea levels than survivors, supporting the notion that blood urea may be indicative of poor COVID‐19 prognosis. Another study by Wang [11] found elevated BUN was independently linked to higher mortality among hospitalized COVID‐19 patients, Patients with increased BUN above 41.0 mmol/L should therefore be closely monitored to improve care outcomes in COVID‐19 patients. A number of conditions, such as dehydration, sepsis, or multiorgan failure, can result in kidney damage or reduced renal function, which is frequently indicated by elevated BUN values. According to Goyal et al. [48], high urea levels can thus be a biomarker for kidney injury which has been noted in critically sick COVID‐19 patients. For COVID‐19 patients, especially those in critical care, monitoring urea levels is essential for spotting early indicators of renal impairment and directing therapy choices. In situations with severe COVID‐19, early identification and treatment of renal damage, including the use of suitable fluid and electrolyte therapy, may improve results [49].

Severe patients recorded significantly low sodium (116.4 ± 14.24 mmol/L) and potassium (5.138 ± 0.7658) levels. The severity of the COVID‐19 disease was shown to considerably lower the mean blood sodium and potassium levels in this investigation. Hyponatremia has been linked in studies to more severe illness, longer hospital admissions, and higher death rates. According to research, individuals who had severe hyponatremia (< 125 mmol/L) had poorer outcomes, especially if they needed intensive care [50]. It has been frequently documented that electrolyte abnormalities, particularly potassium and sodium imbalances, are frequent clinical symptoms of COVID‐19 [51]. We observed significantly lower potassium levels in the severe patients as compared to the asymptomatic and moderate individuals. Studies show that about 20%–30% of hospitalized COVID‐19 patients exhibit low potassium levels [52]. Important pathophysiology of hypokalemia in COVID‐19 includes anorexia attributable to severe acute illness or comorbidities, gastrointestinal loss due to diarrhea, kidney loss due to diuretic usage or tubular damage from ischemia or nephrotoxic chemicals, and gastrointestinal loss due to use of diuretics [53]. A systematic review and meta‐analysis by Liu et al. [54] suggests that individuals with acute myocardial infarction who had lower (3.5 mEq/L) or higher (4.5 mEq/L) blood potassium levels were at higher risk of death. A comprehensive retrospective study found that individuals with the lowest mortality had mean potassium concentrations between 3.5 and 4.0 mmol/L [55]. Furthermore, a high normal potassium level was linked to a poor prognosis and a higher mortality rate; this can be explained by the presence of metabolic and respiratory acidosis with extracellular potassium shifting [56]. The significance of electrolyte monitoring and control as part of the overall care plan is highlighted by the reported decreases in sodium and potassium levels among severe COVID‐19 patients. Respiratory failure, circulatory instability, and renal dysfunction are all frequent consequences of severe COVID‐19 that can be made worse by hyponatremia and hypokalemia [57]. The wider systemic consequences of the virus, such as abnormalities in fluid balance, renal function, and hormone control, may also be reflected in these electrolyte abnormalities [58].

Our study had some limitations. Only a small number of hematological, inflammatory, and biochemical biomarkers were investigated due to budget constraints. Furthermore, potential confounders such as obesity and drug use were also not reported thus source of bias. Additional biomarkers such as D‐dimer, ferritin, IL‐6, and pro‐lactonin could better inform on the levels of inflammatory biomarkers in SARS‐CoV‐2 patients. Secondly, a small sample size was used in this study, thus may lack wider generalizability. Third, the study's generalizability to specific populations at higher risk for severe COVID‐19 may be limited as the study excluded pregnant women. Lastly, the comorbidities were self‐reported and thus may not have been accurate. This will also contribute to developing a solid plan to control and reduce the pandemic.

In conclusion, our research shows that serum CRP and LDH levels are important markers of COVID‐19 severity and progression. Studied hematological indices and biochemical markers at admission can help predict severity inform appropriate management and help alleviate high COVID‐19 mortality. To quickly identify COVID‐19 individuals who are more likely to have severe disease, clinicians can employ inflammatory and hematological biomarkers. This will allow for earlier intervention to reduce complications.

## Author Contributions

**Rebeccah M. Ayako:** conceptualization, data curation, formal analysis, investigation, methodology, project administration, visualization, writing–original draft, writing–review and editing. **Kirtika Patel:** funding acquisition, investigation, project administration, writing–review and editing. **Isaac Ndede:** conceptualization, formal analysis, supervision, validation, writing–review and editing. **Johan Nordgren:** conceptualization, formal analysis, methodology, software, writing–review and editing. **Marie Larrson:** conceptualization, formal analysis, supervision, validation, visualization, writing–original draft. **Simeon K. Mining:** conceptualization, project administration, supervision, writing–review and editing.

## Conflicts of Interest

The authors declare no conflicts of interest.

## Data Availability

The data that support the findings of this study are available on request from the corresponding author. The data are not publicly available due to privacy or ethical restrictions.
